# A Case of Associated Laryngeal Paralysis Caused by Varicella Zoster Virus without Eruption

**DOI:** 10.1155/2014/916265

**Published:** 2014-03-12

**Authors:** Keishi Fujiwara, Yasushi Furuta, Satoshi Fukuda

**Affiliations:** ^1^Department of Otolaryngology, Head and Neck Surgery, Graduate School of Medicine, Hokkaido University, Kita-Ku, N15W7, Hokkaido, Sapporo 0608638, Japan; ^2^Department of Otolaryngology, Head and Neck Surgery, Teine-Keijinkai Hospital, Sapporo 0068555, Japan

## Abstract

We report a patient with significant weakness of the left soft palate, paralysis of the left vocal cord, and left facial nerve palsy. Although the patient showed no herpetic eruption in the pharyngolaryngeal mucosa and auricle skin, reactivation of varicella zoster virus (VZV) was confirmed by serological examination. She was diagnosed with zoster sine herpete. After treatment with antiviral drugs and corticosteroids, her neurological disorder improved completely. When we encounter a patient with associated laryngeal paralysis, we should consider the possibility of reactivation of VZV even when no typical herpetic eruption is observed.

## 1. Introduction

Ramsay Hunt syndrome (RHS) is caused by reactivation of varicella zoster virus (VZV) [1]. Typically, RHS is characterized by zoster oticus, facial nerve palsy, and cochleovestibular symptoms. However, eruption is not observed in all cases, with the cases not presenting with eruption being defined as zoster sine herpete (ZSH) [2]. Distinguishing ZSH from Bell's palsy can be difficult. To diagnose ZSH accurately, detection of VZV DNA by polymerase chain reaction (PCR) or a significant elevation in serum anti-VZV antibody titer using a complement fixation test or enzyme-linked immune sorbent assay (ELISA) is needed.

Associated laryngeal paralysis is a clinical condition merged with other cranial nerve disorders associated with vocal cord paralysis. Recently, it has been speculated that a proportion of idiopathic associated laryngeal paralysis are due to VZV [3]. Most cases might be misdiagnosed as idiopathic laryngeal paralysis due to lack of appropriate serological tests. Although there are some reports on laryngeal zoster and associated laryngeal paralysis with mucosal eruption [4, 5], cases of associated laryngeal paralysis due to VZV without eruption are rare [6]. Here, we report a case of ZSH presenting with associated laryngeal paralysis and facial nerve palsy.

## 2. Case Presentation

**Figure 1:**
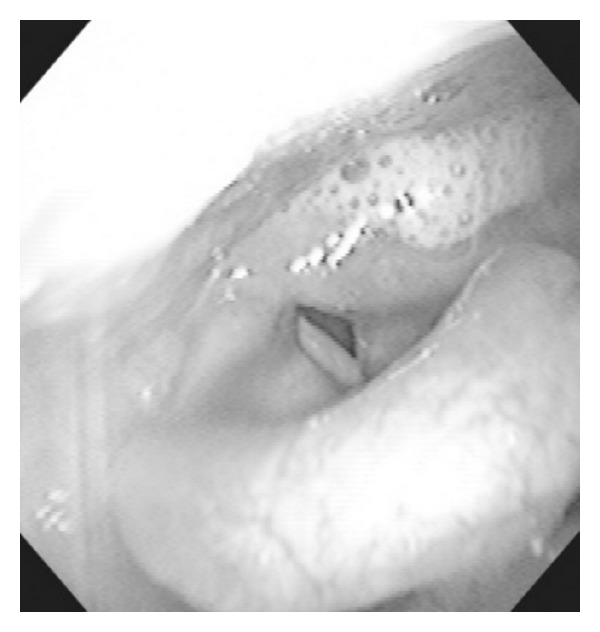
Laryngoscopic examination. Laryngoscopy revealed saliva pooling in the left piriform sinus. No eruption was observed in the pharyngolaryngeal mucosa.

A 50-year-old female presented with hoarseness, dysphagia, and left ear pain on February 10. She was diagnosed with a common cold and received medication from a clinic. Her symptoms did not improve and she visited our hospital on February 14. Physical examination demonstrated significant weakness of the left soft palate with a deviation to the right side during phonation. There was no sign of herpetic eruption within the region surrounding her ear, face, and oral cavity. Her facial sensation was normal and no facial weakness was observed. Laryngoscopy revealed paralysis of the left vocal cord and saliva pooling in the left piriform sinus (Figure 1). There were no vesicles or erosion on the pharyngolaryngeal mucosa. She showed normal hearing level by pure tone audiometry and her stapedius reflex was normal. No nystagmus was observed. Magnetic resonance imaging (MRI) showed no lesions of brain stem.

At this point, the diagnosis was idiopathic associated laryngeal paralysis. However, we considered the possibility of VZV reactivation from the presence of facial discomfort and ear pain, in spite of the lack of typical skin rash or mucosal eruption. The patient was admitted to our hospital and treatment with antiviral therapy and a short course of oral corticosteroids was started according to our therapeutic strategy for RHS. Seven hundred fifty mg of intravenous acyclovir per day for a week and 60 mg of prednisolone per day for 5 days were administrated and the dose was tapered. On the second hospital day, she complained of left facial weakness, and mild facial nerve palsy with House-Brackmann (HB) grade II was observed. Eight days later, the soft palate and vocal cord were beginning to show movement, the hoarseness was improving, and the facial nerve palsy showed no change from HB grade II. Three weeks later, her facial weakness was improved. Paralysis of the soft palate and vocal cord was completely resolved six weeks later.

On the first and 15th day, ELISA for VZV and herpes simplex virus (HSV) was undertaken as HSV can also produce lower cranial nerve palsies. Although ELISA for VZV IgM was negative at the first examination, a slight elevation was observed in the paired sera (1.10, negative <0.80). Results for VZV IgG showed a significant increase from 10.5 to >128. From these results, we made a diagnosis of reactivation of VZV. Both the first and paired sera were negative for HSV IgM and IgG.

## 3. Discussion

A significant elevation in VZV antibody titers is observed in some of the patients diagnosed as idiopathic associated laryngeal paralysis [3]. It is not rare for samples to be negative for VZV reactivation at the first examination, and significant changes (greater than 2-fold) in IgG antibody values in paired sera are considered to indicate a recent VZV infection [7]. VZV IgM has been reported to be found in the acute phase in only 11% of patients with herpes zoster [8]. From a significant elevation in VZV IgG observed in the paired sera, the cause of associated laryngeal paralysis was thought to be reactivation of VZV. We finally diagnosed this case as ZSH because of the lack of typical eruptions seen in laryngeal zoster. We cannot exclude the possibility that the eruption disappeared before her initial visit. Recently, VZV is one of the most important causes of laryngeal paralysis and paralysis due to VZV without eruption is not so rare. However, most cases might be misdiagnosed as “idiopathic” due to lack of appropriate serological tests.

In this case, facial nerve palsy followed associated laryngeal paralysis. In patients of RHS, VZV reactivation occurs in the geniculate ganglion and the subsequent neuritis may result in facial nerve palsy [9]. On the other hand, in cases of lower cranial polyneuropathy caused by VZV infection, as in this case, inflammation may occur in the nuclei of the affected nerves. Generally, the prognosis for facial nerve palsy caused by VZV reactivation is poorer than that of Bell's palsy [10]. But, in this case, only mild facial nerve palsy of HB grade II was observed and the palsy was resolved within a relatively short period of time. There are two possible explanations. One is that treatment by antiviral drugs before the development of facial nerve palsy might reduce inflammation in the facial nerve. The other is that the main part of the inflammation in this case might have begun in the glossopharyngeal or vagal ganglion, before spreading to the facial nerve.

In patients with RHS and ZSH, the early administration of antiviral drugs improves the prognosis of facial nerve palsy [10–12]. The value of early antiviral treatment may be similar in patients with associated laryngeal paralysis caused by VZV reactivation [3]. If unilateral eruption in the pharyngolaryngeal mucosa is observed in patients with associated laryngeal paralysis, the possibility of VZV reactivation should be considered. However, in cases without herpetic eruption, serological examination or detection of VZV DNA by PCR from saliva is needed to diagnose VZV reactivation. As paired sera are needed for diagnosis by serological examination, it takes 2-3 weeks until reactivation is confirmed [7]. Although the results of PCR can be obtained quickly, PCR is not easily carried out in all hospitals. Interestingly, Kakizaki reported six cases of laryngeal herpes zoster, including two cases of ZSH, and found that all patients complained of pain such as a sore throat or pain on swallowing [6]. In cases of associated laryngeal paralysis with such pain, VZV infection should be considered as a differential diagnosis. If the antiviral therapy is administered early, a better prognosis might be obtained in cases of associated laryngeal palsy due to VZV without eruption. It is hoped that a method of early diagnosis for cases of VZV reactivation can be established in the future.
